# Ontologies in Quantitative Biology: A Basis for Comparison, Integration, and Discovery

**DOI:** 10.1371/journal.pbio.1000374

**Published:** 2010-05-25

**Authors:** Lars J. Jensen, Peer Bork

**Affiliations:** 1 Novo Nordisk Foundation Center for Protein Research, Faculty of Health Sciences, University of Copenhagen, Copenhagen, Denmark; 2 European Molecular Biology Laboratory, Heidelberg, Germany; 3 Max Delbrück Center for Molecular Medicine, Berlin-Buch, Germany

## Abstract

As biology is becoming a data-driven discipline, ontologies become increasingly important for systematically capturing the existing knowledge. This essay discusses current trends and how ontologies can also be used for discovery.

Biology is rapidly changing from a descriptive to a data-driven discipline in which the discovery of novel findings depends on the comparison and integration of massive data sets. As a consequence, ontologies—systematic descriptions of specific biological attributes—are becoming more and more important for describing the existing biological knowledge. Despite an increasing awareness about ontologies among biologists, much work remains to be done before many research fields in biology can benefit from capturing the knowledge in such a way. We explore, here, the use of biological ontologies, illustrate how ontologies can be used to make discoveries, and discuss some of the challenges to using ontologies for more than descriptive purposes.

The word *ontology* is derived from Greek öντος (of being) and -λογία, (study) and refers to the philosophical study of the nature of being and existence. In computer science, an ontology is an explicit specification of a conceptualization that defines the objects, concepts, and other entities that are presumed to exist in some area of interest and the relationships that hold among them [1].

Ontologies have a long tradition in biology and medicine, although many of them are normally referred to as taxonomies or classifications. A very early example of a biological ontology is the Linnaean taxonomy from the mid 1700s, which describes relations between species and, combined with the work of Charles Darwin, forms the basis for modern taxonomy and our understanding of evolution. Today, ontologies have become an essential part of modern molecular biology, enabling large-scale comparison, integration, and sharing of data. Many of the early ontologies are usually not thought of as ontologies or formally specified in ontological terms, yet they form the conceptual basis of molecular biology. For example, they focus on classification of and relationships between biological entities or concepts such as amino acids [2] and protein structures [3],[4], protein and domain families [5]–[7], as well as associated molecular functions [7],[8] and biochemical pathways [9].

## The Rising Awareness About Biomedical Ontologies

**Figure 1:**
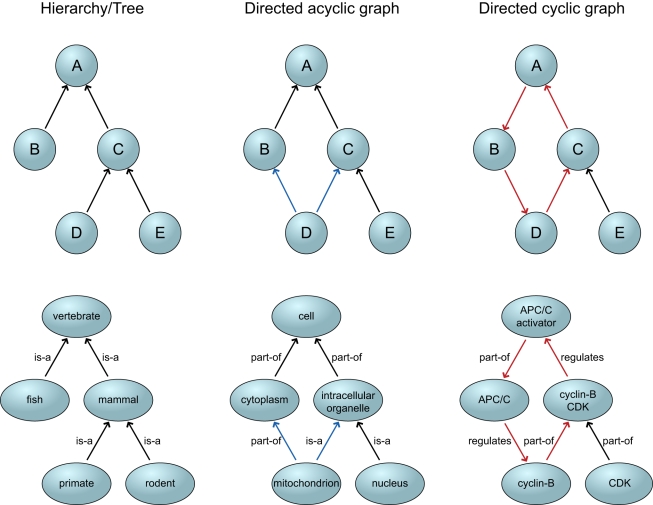
Typical structures of ontologies. Almost all biomedical ontologies are either simple tree structures that represent hierarchical classifications or directed acyclic graphs (DAGs). The difference is that the latter allows a term to be related to multiple broader tems (green arrows) whereas the former does not. Directed cyclic graphs are very rarely used for ontologies; the reason is that cycles (red arrows) can only arise in ontologies that make use of other relationships than *is-a* and *part-of* are used [28]. We illustrate each structure with simplified examples, namely an ontology of vertebrates, an ontology of cellular components, and an ontology of cell-cycle regulation that shows the mutual regulation of cyclin-dependent kinase (CDK) and anaphase-promoting complex/cyclosome (APC/C).

The ontologies mentioned above are all fairly simple classification schemes that consist of either a single level of categories or a hierarchy of categories (i.e., a tree structure). One of the most well-known ontologies, Gene Ontology (GO), integrates model organism databases to provide descriptions of gene products across organisms using standardized, machine-readable language. To tackle the problem of describing protein functions in their cellular context, GO uses a more complex structure known as directed acyclic graph (DAG) [10]. The difference between a tree and a DAG is that in the latter a term can be related to multiple broader terms rather than only one (Figure 1). This allows GO to elegantly model, for example, that *receptor tyrosine kinases* are both *receptors* and *kinases*.

**Figure 2:**
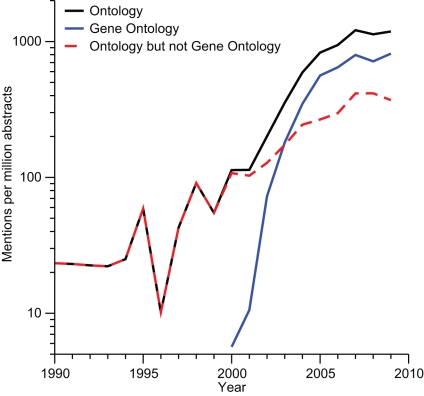
The growth of ontologies in biomedicine. To illustrate the increasing use of ontologies, we mined PubMed abstracts for occurrences of the words *ontology* and *gene ontology* (and the plural forms thereof). We normalized for the general growth of PubMed by converting the raw counts per year to “hits per million abstracts.” The plot shows a steady increase in the awareness of ontologies over the past decade, and that GO became the dominating biological ontology over a period of just five years (note the logarithmic scale). However, ontologies appear to have reached a plateau over in the past three years, at least in terms of how often they are mentioned in abstracts. In contrast, the citations to GO and associated resources are steadily rising (end of 2009>5500) and imply a further increasing use.

GO has had a major impact on the awareness and use of ontologies in biology. Prior to publication of GO in 2000 [10], less than one in 10,000 new abstracts added to Medline would mention the words *ontology* or *ontologies*. By 2007 that number had increased by more than an order of magnitude, and more than two thirds of the abstracts that mention ontologies specifically mention GO (Figure 2), which is also reflected in the steady rise in the number of the citations to GO and associated resources. This can, in part, be attributed to the use of GO within rapidly growing research areas such as comparative genomics, transcriptomics, and proteomics. Another important contribution is that the GO consortium worked closely together with the communities behind key model organism databases to ensure that vast amounts of GO-based annotations would be provided for each of the respective genomes.

An ontology is much like a database: until it is used to organize actual data, it is an empty shell that is not of much use to anyone. Once large amounts of data are easily accessible in a structured form, numerous tools will almost certainly be designed to make use of it. Indeed, half of the top 10 cited papers related to the topic *ontologies* present tools that allow easy statistical analysis and inspection of GO terms for a set of genes or proteins, for example, identified in a transcriptomics or proteomics study [11]–[15]. As a direct consequence, GO is one of the few ontologies that are frequently used to describe and compare large-scale datasets and have succeeded in revealing trends that might otherwise have been overlooked. For example, GO analysis suggested that translational repression is stronger for mRNAs translated by endoplasmic-reticulum-associated ribosomes compared to free cytosolic ribosomes [16]. Going beyond convenient overviews and utilizing ontologies to reveal new biological insights remains one of the major challenges. So far, most efforts to enhance ontologies are directed at broadening the biological knowledge that is formalized using ontologies.

Recently, interaction ontologies have been developed that complement the current GO scheme on functional classifications. These ontologies aim to describe the types of interactions that can take place between biomolecules, including binding, regulation, and modification [17]–[20]. GO has taken the first small step in this direction by adding new relationship types related to regulation [21].

Numerous other biomedical ontologies are being developed that are useful for providing context for the functions of genes, proteins, and small molecules. Furthermore, ontologies cover complex biological processes and systems such as anatomy, development, and phenotypes. In medicine, ontologies such as Medical Subject Headings (MeSH), International Classification of Diseases (ICD), Systematized Nomenclature of Medicine–Clinical Terms Diseases (SNOMED-CT), Anatomical Therapeutic Chemical (ATC) classification system, and Coding Symbols for Thesaurus of Adverse Reaction Terms (COSTART) are extensively used to classify diseases, symptoms, drugs, and side effects. Currently, about 200 biomedical ontologies are listed in databases like http://www.bioontology.org/ and http://www.obofoundry.org/. Yet, there are obvious areas such as the interaction of species with the environment (e.g., lifestyles and habitat similarities) where first attempts have been made (http://www.environmentontology.org), but which deserve more attention in the future.

## Using Ontologies for Discovery

In addition to having important roles in genome annotation and statistical characterization of gene sets, ontologies have the potential to help scientists make new discoveries. To our knowledge, this potential has only been realized in a few case studies so far.

An early example is the Genes2Diseases method [22], which predicts candidate genes for inherited diseases in a given locus by correlating molecular functions of the genes therein assigned by GO with controlled vocabularies in chemistry, diseases and phenotypes provided by the Medical Subject Headings in Medline (MeSH terms). For example, the glutamate dehydrogenase 1 and 2 genes, which reside within the linkage region of the disease *spinocerebellar ataxia-8, infantile, with sensory neuropathy*, were predicted to be involved in the disease based on literature links between the GO term *glutamate catabolism* and the MeSH term *spinocerebellar degenerations*.

**Figure 3:**
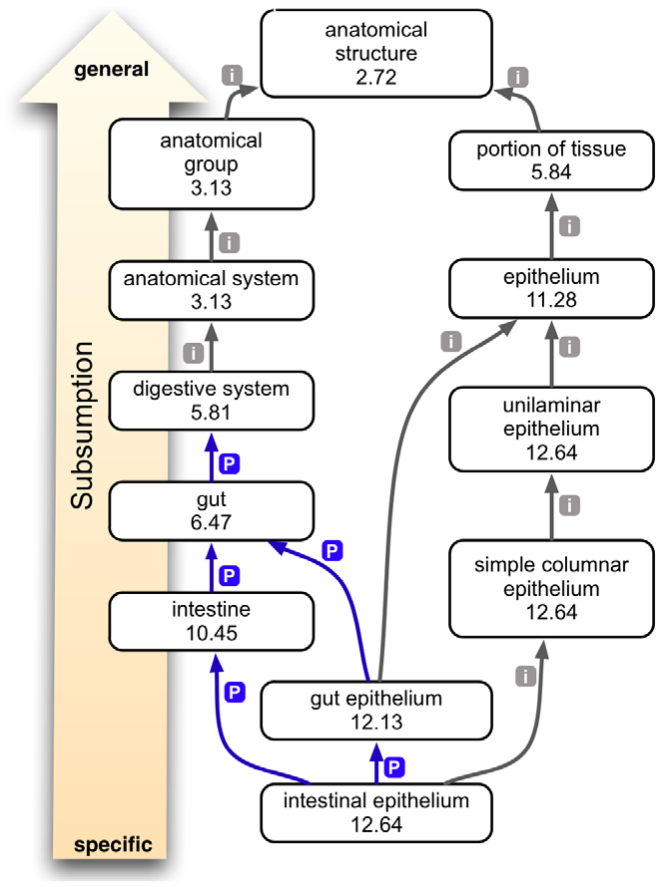
Ontology subsumption reasoning. This example from Washington et al. [23] shows the relationships of the term “intestinal epithelium” to other anatomical entities within the ZFA ontology. Gray arrows with an “i” indicate an is-a relation, and blue arrows with a “p” indicate a part-of relation. The numbers indicate IC of the node, which is the negative log of the probability of that description being used to annotate a gene, allele, or genotype (collectively called a feature). As terms get more general, reading from bottom to top, they have a lower IC score because the more general terms subsume the annotations made to more specific terms.

Recently, two new uses of ontologies for discovery have been published. The first links human diseases to animal models through cross-species comparison of phenotypes and anatomical structures [23], and the second identifies hitherto unknown targets for existing drugs by comparing side effects [24]. Despite the completely different goals, the two methods conceptually have much in common: concepts (be they diseases, animal models, or drugs) are linked based on the similarity of the sets of phenotypes that they are associated with. The phenotypes are described using terms from ontologies, which are backtracked to broader, parental terms in the ontology (see Figure 3). Finally, the resulting sets of phenotypes are compared using scoring schemes that take into account the frequencies of the term, because rare terms are generally more informative than very common terms. There is one main conceptual difference between the two approaches. Washington et al. needed to compare phenotypes across species-specific anatomical ontologies to identify genes in model organisms that are phenotypically similar to 11 human diseases [23]. By contrast, Campillos et al. could directly compare the phenotypic side-effect profiles of different drugs, because all side-effect data were directly obtained from human subjects, and thereby identify 261 pairs of chemically dissimilar drugs from different therapeutic indications that are likely to share targets [24].

These approaches go beyond the type of logical reasoning that is usually associated with ontologies. The major difference is that they can identify plausible relationships that are supported by the existing knowledge, but which are not strict, logical consequences thereof [25]. For example, a human disease can be linked to an animal model based on having similar annotated phenotypes. The underlying idea that a computer can discover a new, previously unknown relationship (A–B) based on two or more known relationships (A–C and B–C) is reminiscent of the early (mostly manual) text-mining work by Don Swanson who, for example, correctly predicted that fish oil can ameliorate Raynaud disease based on both concepts being linked to platelet inhibition, vasodilation, lowered blood viscosity and triglyceride levels, increased prostacyclin, and blocking of serotonin release [26]. In other words, the phenotypic profile of the response to fish oil matched that of successful treatments of the disease.

## Current Challenges and Future Needs

The works described in the previous section illustrate the promise of using ontologies for biomedical research, but also highlight some of the many challenges that must be overcome if we are to realize the power of ontologies and to move beyond descriptions to discoveries. Despite basing their work on existing ontologies, both discovery projects involved a considerable investment of time in annotating the current knowledge within the field (domain knowledge) according to the ontologies. Although this annotation process was aided by the use of text-mining tools, domain experts must check all the extracted facts. Quantity and quality of annotation is, as alluded to earlier, the make or break of any ontology.

A prerequisite for making good annotations is that the human annotators use the terms from the ontology consistently. It is thus crucial to have clear definitions of all the terms. The sequence ontology (SO) is so far the only biological ontology to formally specify the meaning of terms [27]. Although formal definitions are certainly more stringent than the textual descriptions that all other biological ontologies rely on, we believe that the latter are more important for ensuring consistency of how the terms are used by annotators.

Widely used ontologies like GO tend to run into a second problem that only enhances the former one: concept explosion. GO currently consists of over 30,000 different terms, which makes it nearly impossible for a human annotator to know all terms, their precise definitions, and how terms relate to each other. For example, one could easily be mistaken to think that the GO term *DNA replication* would automatically imply the term *cell cycle*, but that is not the case because some DNA replication proteins are used only in the mitochondria or in response to DNA damage. Nonetheless, there are many examples of cell-cycle genes in the model organisms that are annotated with the former GO term but not the latter, which simply shows that even the best database curators cannot be expected to memorize a DAG of more than 30,000 terms. Whereas complex ontologies allow more fine-grained annotations to be made, we suspect that simpler ontologies may lead to fewer mistakes.

GO's success can partly also be attributed to its consistent use of the same ontology across species, which facilitates simple similarity-based function annotation and cross-species comparisons. However, it is a major challenge to accommodate species-specific differences within a single ontology. This is especially true when dealing with concrete physical entities; for example, budding yeast and humans do not have the same complement of protein complexes, which makes it difficult to define a unified set of protein complexes within the GO cellular component ontology. This issue only becomes more difficult when dealing with anatomy or developmental stages, for which species-specific ontologies are currently used. In such cases the way forward may be to bridge the species-specific ontologies by identifying orthologous genes, that is, genes from different species with common ancestry, as was done by Washington and colleagues [23].

The two largest challenges, though, are that the vast majority of the biological knowledge currently exists only as unstructured text, and that much of what is described in a typical publication cannot be captured by current ontologies. At best, current ontologies describe the type of information that would be given in the Conclusions section of a paper. However, the most important details typically reside in the Results and Methods sections: which observations were made and under which exact conditions. To capture this in a structured form, which will be crucial for interpreting the future flood of experimental data, we will need ontologies such as the Ontology for Biomedical Investigations (http://www.obi-ontology.org) that are more closely tied to the experiments and measurements themselves, rather than to the researchers' interpretations thereof. Maintaining such ontologies will be particularly difficult because new experimental techniques are continuously developed, making a comprehensive description of them a moving target. Even with such ontologies in place, the challenge remains to get the information described according to ontologies. This will likely require the development of tools that will help authors annotate the text as they write it, and possibly readers to subsequently improve the annotations as the ontologies themselves are expanded (Box 1).

**Box 1. Semantic Annotation of Scientific Publications**

To capture the knowledge of a publication in computer-readable form, the text must somehow be semantically annotated, that is, the meaning of the text must be described using standardized names and terms.

**Why?** The biomedical literature is growing exponentially, and we are already past the point where it is impossible to read all papers published on topics such as the cell cycle [29],[30]. Reading thus needs to be supported by semantically enhanced literature and ontology-aware tools that provide computational access to the underlying knowledge [30].

**What?** It is unclear how much of the meaning of an article should be captured by semantic annotation. Although more is always better, it is important to keep in mind that anything is better than nothing. We should thus already now start to annotate the genes, proteins, functions, interactions, and phenotypes mentioned in each publication with their respective database identifiers and ontology terms. The scope of semantic annotations should subsequently be gradually extended as new ontologies are developed.

**Who?** One option is to have authors annotate their manuscripts, since they know better than anyone exactly what was meant. However, one cannot expect authors to be sufficiently well versed in ontologies to be able to make all the applicable annotations, for which reason they may need support from database curators. The latter could also contribute annotations directly; it would be desirable that the large effort that goes into constructing biological databases would also improve the annotation of the underlying literature. Finally, one could allow readers to add and correct annotations.

**When?** One can envisage several points during the life cycle of an article when annotations could be added. Authoring tools could help researchers annotate the text with appropriate ontology terms while writing the manuscript, which as an attractive side-effect would encourage consistent usage of scientific terms in the text itself. Semantic annotation could alternatively become part of turning an accepted manuscript into a publication. However, the annotation process need not end at the time of publication; readers could correct erroneous or missing annotations and extend the scope of annotation in already published articles as new ontologies are developed.

**Where?** There are several options as to where the semantic annotation of a publication could be stored. One option is to embed it directly in the documents. This ensures the tightest possible link between text and annotation but would force the annotations to be static, unless one allows post-publication changes to documents. Alternatively, annotations could be stored in centralized databases operated by publishers or public information centers, or in a distributed manner that leaves the individual content consumers to decide which types and sources of annotations to include.

The answers to these questions are obviously not independent of each other, and the alternative approaches are complementary rather than mutually exclusive. Several different approaches have already been tested, often in collaboration with publishers, and it is clear that tools will be required to ease the burden of manual annotation by suggesting semantic annotations where appropriate (see [31] and references within).

The need for computer-readable ways to express our knowledge is closely tied to the exponential growth in biological data. Human-readable textual descriptions suffice when analyzing only a few genes or proteins, but computer-readable ontologies are a prerequisite for systematic and comparative analysis of whole genomes, transcriptomes, or proteomics. Despite these challenges, the rapid move towards quantitative biology will thus likely drive the development of new biological ontologies. The sheer number of high-throughput experiments implies that ontologies will be needed for describing not just the genes and proteins but also information about the samples and experiments themselves (i.e., metadata). Finally, current ontologies are qualitative in nature, whereas ontologies that can capture quantitative knowledge (e.g., fold-changes) in time, space, and context (e.g., environmental factors) will be needed to fully describe the conclusions derived from quantitative experiments.

## Abbreviations

DAG: directed acyclic graph
GO: gene ontology
MeSH: Medical Subject Headings
